# Predicting Suitable Habitat for *Glipa* (Coleoptera: Mordellidae: Mordellinae) Under Current and Future Climates Using MaxEnt Modeling

**DOI:** 10.3390/insects16060642

**Published:** 2025-06-18

**Authors:** Xie Su, Xianheng Ouyang, Xiaoqun Ding, Yang Wang, Wangang Liu, Yang Liu

**Affiliations:** 1 Key Laboratory of Resource Biology and Biotechnology in Western China, College of Life Science, Northwest University, Taibai North Road 229, Xi’an 710069, China; mtghfs@163.com (X.S.); xiaoqun_d@163.com (X.D.); 2 College of Forestry, Northwest A&F University, Yangling 712100, China; oyxh@nwafu.edu.cn; 3 Shangluo Research Center of Chinese Medicinal Materials Integrated Pest Management, Shangluo University, Shangluo 726099, China; wyang369@163.com; 4 State Key Laboratory of Loess Science, Institute of Earth Environment, Chinese Academy of Sciences, Xi’an 710061, China; 5 Department of Entomology, University of Manitoba, Winnipeg, MB R3T 2N2, Canada

**Keywords:** MaxEnt, climate change, habitat suitability, biodiversity conversation

## Abstract

Beetles of the family Mordellidae, important global pollinators, include *Glipa*, the third largest genus, which retains plesiomorphic traits related to pollination and is mainly found between 38° S–38° N. Existing studies on *Glipa* focus largely on taxonomy and systematics. The ecological response of *Glipa* to climate change remains poorly understood. Our objective was to investigate how the distribution of *Glipa* may respond to climate change using a species-level MaxEnt based model with 297 geographic distribution data points and seven bioclimatic environmental variables. The study showed that the MaxEnt model had a high predictive accuracy, with an Area Under the Curve (AUC) value of 0.963. The maximum temperature of the warmest month, mean annual precipitation, and mean precipitation of the driest quarter were the three most important factors affecting the distribution of *Glipa*. Currently, the suitable distribution areas of *Glipa* are mainly located in East Asia, Southeast Asia, eastern North America, South America, and central and western Africa. Under future climate scenarios, the area of suitable habitat is expected to increase gradually as global temperatures rise. Under the SSP585 scenario in the 2070s, the suitable habitat area is projected to expand by 53.89% compared to the present. Additionally, the centroid of suitable habitat is expected to shift northward. This study not only deepens the understanding of the distribution patterns of *Glipa* and their response to climate change but also provides important scientific evidence for the conservation of pollinator diversity.

## Simple Summary

Based on 297 geographic distribution records and seven bioclimatic variables, this study predicted the potential distribution of *Glipa* under current and future climate change scenarios by using the MaxEnt model (v3.4.4) and ArcGIS (v10.8). The results of the study show that the maximum temperature of the warmest month, mean annual precipitation, and mean precipitation of the driest quarter were the three most important factors affecting the distribution of *Glipa*. Currently, *Glipa* is primarily distributed in tropical and subtropical regions across East and Southeast Asia, eastern North America, South America, and parts of central and western Africa. Under future climate scenarios, the area of suitable habitat is expected to increase gradually as global temperatures increase. The study provides a theoretical basis for the conservation of pollinator diversity in the context of climate change.

## 1. Introduction

Climate change is already causing widespread effects on ecosystems at the global level and is predicted to intensify over the next 20 years [1]. The global mean surface temperature (GMST) will increase by at least 1.5 °C in the short term (2021–2040), a rate double or more than the temperature increase observed over the past 100 years [2]. If global warming ranges between 1.5 °C and 2 °C, the risk of extinction for endemic species in biodiversity hotspots is predicted to rise from 2% to 4%. This risk will increase tenfold if the temperature increase reaches 3 °C [2]. Climate change is one of the primary drivers of biodiversity degradation, which leads to the relocation and extinction of local species and ecosystem instability [3].

Pollination is an important process in most terrestrial ecosystems, which plays a vital role in maintaining ecosystem stability and conserving biodiversity [4]. Approximately 88% of angiosperms depend on animal pollinators, and this proportion rises up to 94% in terrestrial tropical ecosystems [5]. Insect pollination currently accounts for more than 80% of global pollination, and it is estimated that the total number of pollinating insect species worldwide is approximately 3.5 × 10^5^ [6,7]. Pollination services provided by insects are vital to human sustenance, especially in food production, and provide essential services by increasing agricultural productivity and maintaining agroecosystem stability [8,9]. Insect pollinators contribute to approximately 35% of global food production [8]. Moreover, the pollination services provided by these insects add over $200 billion in economic value to global agriculture annually, according to the United Nations.

Climate change may exacerbate an overall reduction in insect populations, as well as affect host plant phenology, resulting in a desynchronization between the activity of adult insect pollinators and plant flowering phenology [10]. Such changes may ultimately reduce the efficiency and quality of pollination services provided by insects and, hence, ecosystem stability [11]. Desynchronization between insect emergence and plant flowering may drive shifts in species distribution, as insects track suitable phenological windows under changing climates. In this context, understanding the pollination traits of the *Glipa* species is critical. The *Glipa* species are generalist pollinators capable of visiting a wide range of flowering plants, which may confer some resilience to environmental changes. However, even generalists may suffer reduced pollination efficiency and range shifts if their activity becomes severely mismatched with key floral resources. Under the pressures of rapid human activities and climate change, hymenopteran pollinators—particularly honey bees and bumble bees—have attracted significant research attention in recent years [6]. In contrast, despite the ecological importance and high diversity of coleopteran pollinators, studies focusing on this group remain relatively scarce [7].

The family Mordellidae, a relatively species-rich group of beetles distributed across all continents except Antarctica, includes 1500 to 2308 recorded species worldwide, but its species richness still falls short of that of other diverse beetle families such as the weevils (Curculionidae), rove beetles (Staphylinidae), ground beetles (Carabidae), leaf beetles (Chrysomelidae), or darkling beetles (Tenebrionidae) [12]. Adult Mordellidae are often found on flowers with some species being important pollinators of a wide variety of angiosperm species [12,13]. Their larvae mostly feed inside plant stems and can be important agricultural and forestry pests [13]. Mordellid beetles have been angiosperm pollinators for at least 100 million years, with amber-preserved specimens showing evidence for the specialized pollination of true dicotyledons. *Glipa* (Mordellidae; subfamily Mordellinae; tribe Mordellini) is the third largest genus of the Mordellidae [12]. It is the largest genus of the tribe Mordellini, which is characterized by a cuneate body, and a broad triangular terminal segment of each maxillary palpus, with almost equal inner and outer margins and a concave shape in cross-section [12]. All of these traits are considered to be associated with effective plant pollination [14].

The genus *Glipa* is known to include 139 species, primarily distributed in warm and humid regions of the Oriental, Palearctic, Neotropical, and Afrotropical realms [15,16]. Current studies on *Glipa* have mainly focused on new regional species records as well as global taxonomy and systematics, while their responses to climate change remain insufficiently explored [17,18,19]. Climate change may lead to a reduction in suitable habitats, resulting in range contractions and population declines, thereby increasing the risk of extinction [3]. In the context of climate change, it is particularly important to deepen our understanding of key pollinators, including *Glipa* species.

Species Distribution Models (SDMs), also known as Ecological Niche Models (ENMs), are used to predict suitability distribution areas for species by combining their geographic distributions with relevant environmental variables [20]. With the help of rapidly evolving Geographic Information System (GIS) tools, statistical models of distribution areas [21,22] have been widely applied in ecological and conservation studies [23,24]. Several SDM models have been developed, including Maximum Entropy (MaxEnt), Genetic Algorithm for Rule-set Production (GARP) [25], BioCLIM, Ecological Niche Factor Analysis (ENFA) [26], and Generalized Linear Model (GLM) [27]. Among these, the maximum entropy (MaxEnt) model is preferred by researchers for three reasons: (i) a high level of predictive accuracy can be achieved using simple, precise mathematical formulae and parameter settings [28,29], (ii) there is greater predictive power and accuracy than in other SDM models [30], and (iii) there is an ability to handle complex interactions between response and explanatory variables, with a robust output even for small sample sizes [31].

Based on species distribution models, this study predicted the potential suitable habitats of *Glipa* under current and future climate conditions, identified key environmental drivers of its distribution, and assessed the potential impacts of climate change on the centroid shift of suitable habitats, providing important scientific evidence for the conservation of pollinator biodiversity in the context of global climate change.

## 2. Materials and Methods

### 2.1. Geographic Coordinate Acquisition for Glipa

**Figure 1:**
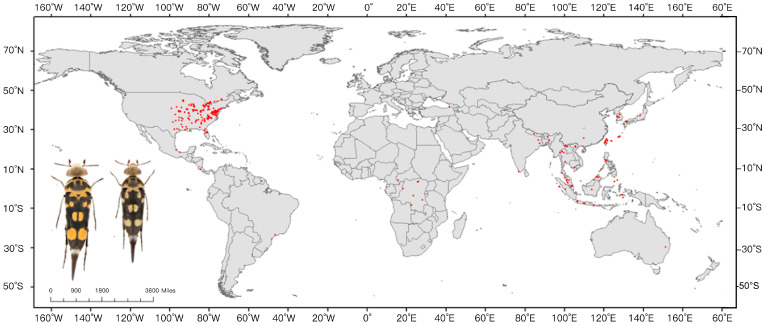
Global Geographic Distribution of *Glipa* based on existing distribution data.

We obtained distribution coordinates from the public database Global Biodiversity Information Facility (GBIF, https://www.gbif.org/ (accessed on 22 December 2022)), and additional relevant literature. Using Google Earth Pro (v7.3.6; Google LLC, Mountain View, CA, USA), we completed distribution records without coordinates. The geographic coordinates of specimens were verified and screened to remove records with vague descriptions or those outside the study area. To avoid overfitting of the MaxEnt model (v3.4.4) caused by clustered data, and considering the spatial resolution of the climate data was 2.5′, only one occurrence point was retained within each 2.5′ × 2.5′ grid cell [32]. Ultimately, 297 valid occurrence points were obtained for constructing the MaxEnt model (Figure 1).

### 2.2. Selection of Environmental Variables

Selecting suitability environmental variables is important for accurate species habitat modeling [33,34,35]. As relevant ecological studies are lacking for most *Glipa* species, we filtered 19 bioclimatic variables from the distribution characteristics of known species and the most commonly used environmental variables in other insect studies (Table S1). The bioclimatic variables data were obtained from the WorldClim Global Climate Database (v2.1), with a spatial resolution of 2.5 arc-minutes. To evaluate future variation in the distribution of *Glipa*, future bioclimatic data were obtained from the National Climate Center (Beijing, China) Climate System Model (BCC-CSM2-MR) of the 6th International Coupled Model Comparison Program (CMIP6). Four shared socioeconomic pathways were included, representing future low- to high-carbon-emission climate scenarios and four periods: 2021–2040 (2030s), 2041–2060 (2050s), 2061–2080 (2070s), and 2081–2010 (2090s) [36].

We conducted Pearson correlation analysis with ENMTools (v1.4.4) to detect potential issues such as autocorrelation and multiple linear repetitions among environmental variables [37]. The 19 bioclimatic variables were pre-modeled with MaxEnt software (v3.4.4), and variables with an absolute correlation coefficient greater than 0.80 were comprehensively screened, considering the importance of variable contribution and the cutter method. Environmental variables that exhibited a higher influence on predictive accuracy of the model, as indicated by high contribution scores, were retained. Conversely, some explanatory variables with high inter-variable covariance were excluded to reduce their influence on simulation prediction results. We selected environmental variables that demonstrated the most significant impact on the species distribution models. This selection was based on the contribution ranking derived from the MaxEnt analysis. Variables that exhibited a higher influence on the model’s predictive accuracy, as indicated by their contribution scores, were retained. Conversely, variables with lower contribution scores or those exhibiting high inter-variable covariance, which could skew the model’s results, were excluded. Seven of the nineteen variables were selected for the final model: Bio2, Bio4, Bio5, Bio12, Bio17, and Bio19 (Table S2).

### 2.3. Model Optimization and Setup

The selection of appropriate model features and regularization coefficients is important for optimizing the model performance [38]. To prevent overfitting and achieve the optimal balance between model complexity and predictive accuracy, we used the ENMeval package (v0.3.1) in R (v4.2.2); R Foundation for Statistical Computing, Vienna, Austria) to optimize the MaxEnt model parameters by adjusting the regularization coefficients and feature combinations [39]. We created a total of 60 alternative models, including 10 regularization coefficients (ranging from 0.5 to 5 with an interval of 0.5) and six different feature combinations (L, LQ, H, LQH, LQHP, and LQHPT, where L denotes linear, Q denotes quadratic, H denotes hinge, P denotes product, and T denotes threshold). To evaluate model performance, we employed a random K-fold cross-validation method (K = 10), and selected the optimal model based on the lowest DAICc value.

We utilized the optimized regularization coefficients and feature combinations to model the potential distribution of *Glipa* using MaxEnt (v3.4.4) [29,40]. When importing species distribution records and environmental variables into MaxEnt (v3.4.4), the preferences selected were as follows: response curves, folded Jackknife test method (measuring variable importance), logical output (output format), random seeds, 10,000 (maximum background points), cross-validation (repeat run type), 10 (repetition number), write graph data and background predictions, and maintained default settings for all other parameters [41]. The predicted distribution results output was in a grid raster format.

### 2.4. Model Accuracy

In order to minimize potential errors in each MaxEnt model, we employed two methods to evaluate accuracy. First, we utilized the area under the receiver operating characteristic (ROC) curve (AUC), which is a commonly used threshold-independent metric in species distribution models (SDMs) [42]. AUC values ranging from 0.9 to 1.0 indicate excellent model performance [43].

### 2.5. Analysis of Suitable Habitats and Their Variation

We analyzed the MaxEnt model results with Excel (v2019; Microsoft Corp., Redmond, WA, USA) and ArcGIS (v10.8; Esri, Redlands, CA, USA) along with its SDMTools toolbox (v2.5). To improve the accuracy of this part of our analyses, the maximum training sensitivity plus specificity threshold was used as the minimum fitness threshold to reclassify and visualize the fitness zone map. Because of fewer influences from species prevalence and background point ratios, the maximum training sensitivity plus specificity threshold (MTSPS) selection method is known for its robustness and can reduce omission errors for low-prevalence species and misclassification errors for high-prevalence species [44]. To describe and reflect the suitable habitat for *Glipa*, we reclassified habitat suitability into four categories: unsuitability (0–0.1416), low suitability (0.1416–0.4), moderate suitability (0.4–0.6), and high suitability (0.6–1.0) [45]. Then, we calculated the potential suitability area for each class based on output logistic values.

To assess the potential spatial distribution and variation of centroids under various climatic scenarios, we converted the model outputs into binary maps using the MTSPS threshold and analyzed the results with SDMTools (v2.5) [46]. Changes in suitable habitat under future climate scenarios were quantified by subtracting current binary maps from binary maps for four future periods, and reducing species distributions to a single centroid.

## 3. Results

**Figure 2:**
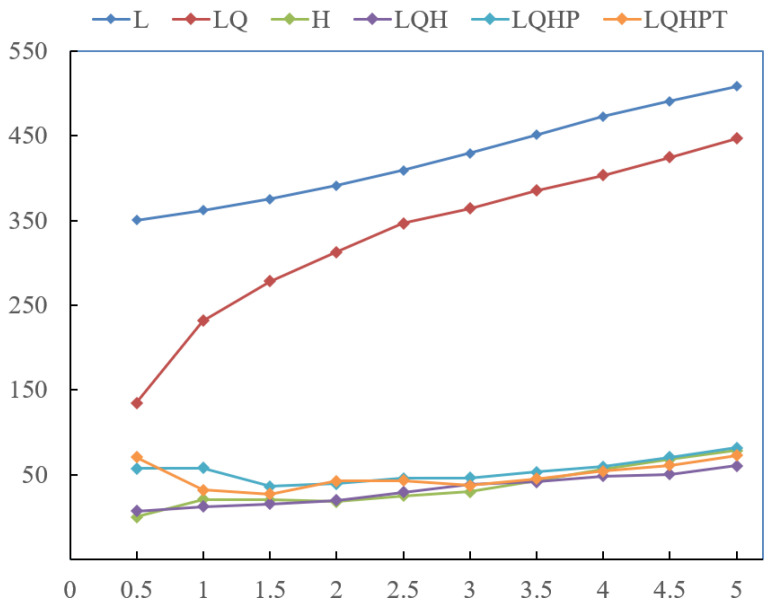
Different characteristic combination results for Glipa by ENMeval of R package.

**Figure 3:**
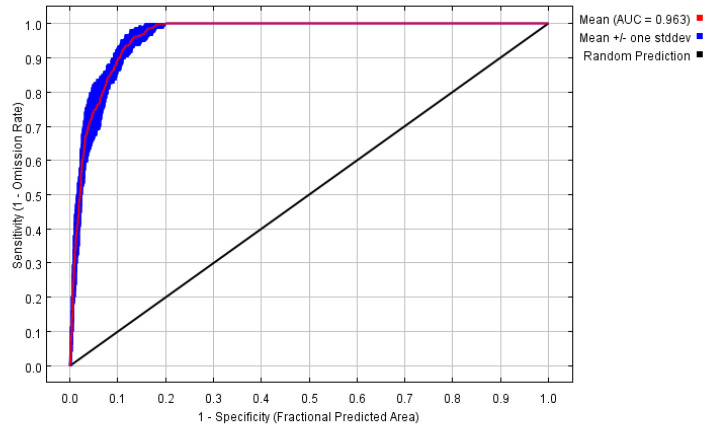
The performance of the optimized MaxEnt model through ROC curve analysis and AUC values for *Glipa*.

Based on these variables and distribution records, we built a predictive model using MaxEnt with FC = H and RM = 0.5 (Figure 2). The model was repeated 10 times, to obtain testing AUC values of 0.963 (Figure 3), respectively, suggesting that the prediction model had excellent predictive performance.

The top three environmental variables ranked by importance were Bio12 (annual precipitation, 44.8%), Bio17 (precipitation of the driest quarter, 27.1%), and Bio5 (maximum temperature of the warmest month, 14.9%), collectively accounting for 86.8% of the total contribution (Table S2). It is noteworthy that Bio12 (annual precipitation) had the most critical independent contribution to predict the global suitable habitats of *Glipa*. Precipitation and temperature were the most important factors, with precipitation having the greater effect.

The environmental variable response curves indicated the extent to which each environmental variable influences the overall model (logistic output), and, thus, habitat suitability for *Glipa* (Figure S1). Logistic values range from 0 to 1, with values close to 1 indicating a habitat of high suitability. When the logistic output exceeded MTSPS, the values of environmental variables were considered to be applicable to *Glipa*. The suitability ranges, optimum values, and highest habitat suitability values for each bioclimatic variable are presented in Table S1. The response curves for Bio2, Bio4, and Bio12 were bimodal: the optimal Mean Diurnal Range (mean of monthly (max temp–min temp)) (Bio2) for *Glipa* was 11.87 °C, with a secondary peak at 3.79 °C (Figure S1a); the ideal Annual Precipitation (Bio12) occurred at 970 mm and 2406 mm (Figure S1d); and the Temperature Seasonality (standard deviation × 100) (Bio4) was most favorable at 0.47 with another peak at 8.88 (Figure S1b). For the remaining four variables, the response curves demonstrated unimodal or near-unimodal patterns: the Max Temperature of Warmest Month (Bio15) was suitable at 3.76 to 88.67, peaking at 11.18 (Figure S1e); the Precipitation during the Driest Quarter (Bio17) suitability for *Glipa* ranged from 78.12 mm to 1094.11 mm, peaking at 244.23 mm (Figure S1f); the Max Temperature of the Warmest Month (Bio5) was suitable from 24.52 °C to 35.84 °C, peaking at 30.64 °C (Figure S1c); and the Precipitation during the Coldest Quarter (Bio19) was suitable from 73.89 mm to 2479.4 mm, peaking at 244.01 mm (Figure S1g).

**Figure 4:**
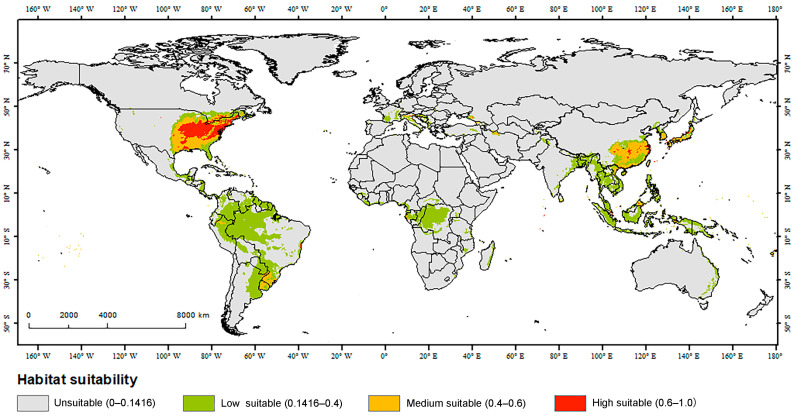
Current potential suitable habitats of *Glipa* on a global scale.

Under the current climatic conditions, the MaxEnt model predicted the potential suitable habitat of *Glipa* (Figure 4; Table S3). The total suitable habitat areas were approximately 2.33 × 10^7^ km^2^. The areas of low, moderate, and high suitable habitats covered 1.76 × 10^7^ km^2^, 0.41 × 10^7^ km^2^, and 0.16 × 10^7^ km^2^, respectively. Suitable habitats were mainly distributed in east Asia, southeast Asia, eastern North America, South America, and central and west Africa. There were also smaller areas of suitable habitat along the Mediterranean coast of Europe and eastern Australia. These results are consistent with the current records, except for western Europe and southern South America along the Atlantic coast, where no *Glipa* have been recorded.

**Figure 5:**
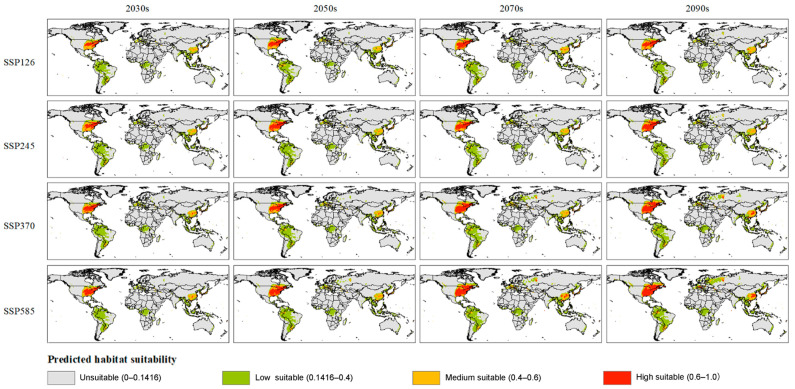
Potential suitable habitats of *Glipa* under different future climate scenarios.

**Figure 6:**
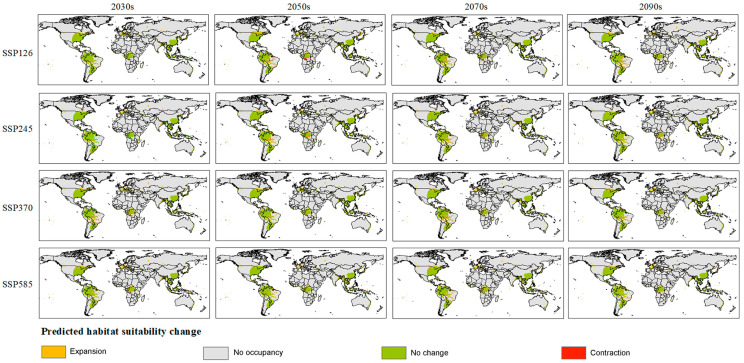
Changes in potential suitable habitat area of *Glipa* under different future climate scenarios.

**Figure 7:**
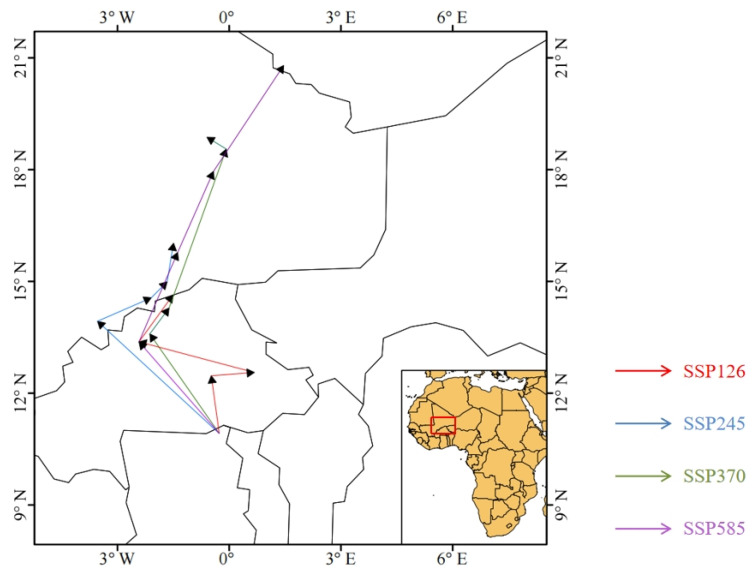
Centroid shift in potential habitat suitability of *Glipa* under different climate scenarios.

Based on filtered important environmental variables, we further simulated the variation of the suitable habitat of *Glipa* under different future climate scenarios (Figure 5, Table S3). Comparing the current suitability range with those predicted in the future, we analyzed the responses of the suitable habitats of *Glipa* to climate change, including expansion, contraction (Figure 6), and centroid shift (Figure 7). Under the SSP585 scenario in the 2070s, the suitable habitat area is projected to expand by 53.89% compared to the current. The global area of suitable habitat was expected to gradually increase, and moderately suitable habitats in the east Asian monsoon region would expand northward in varying degrees. High suitable habitats in eastern North America were expected to expand significantly northwards, with some patchy suitability areas likely to appear in western North America. In Europe, besides the Mediterranean coastal regions, areas of low suitability may appear across eastern Europe in a scattered pattern. In Oceania, Latin America, and Africa, there were no significant predicted changes. Under the SSP5-8.5, SSP3-7.0, and SSP2-4.5 climate scenarios, the centers of suitable habitats will expand toward the northwest and northeast directions, respectively.

## 4. Discussion

### 4.1. Analysis of Environmental Variables

We used the optimized MaxEnt model (implemented with the “ENMeval” package) to identify the bioclimatic factors influencing the distribution of *Glipa* and, then, to predict suitable habitats under current and future climate scenarios. Simultaneously, we compared and assessed the variation in the trends and distribution of suitable habitats for *Glipa*. Temperature and precipitation are often important factors determining insect diversity and distribution [46,47,48]. This is supported by our results, that Bio12, Bio17, and Bio5 all significantly predicted the distribution of *Glipa*. The most suitable habitats for *Glipa* are environments where annual precipitation (Bio12) exceeds ~760 mm, precipitation in the driest quarter (Bio17) exceeds 78 mm, and the maximum temperature during the warmest month (Bio5) is 24.50 °C to 35.80 °C, which is consistent with the localities from which *Glipa* has been recorded [49]. Model predictions indicate that climatically suitable regions such as Western Europe and southern South America currently lack actual *Glipa* occurrence records, which may be influenced by non-climatic factors such as biotic competition, the absence of host plants, geographic isolation, or human activities. We propose that these two factors may influence the distribution of *Glipa* in two ways. First, precipitation and temperature may directly affect the hatching success of eggs, the survival and development of the larvae, and the ecological activity of adults [50]. Second, changes to the precipitation and temperature increase may affect insect distribution and plant–insect interactions indirectly by influencing the patterns of plant phenology [51]. This may destabilize some plant–pollinator mutualisms and, subsequently, adversely affect many additional ecological processes [52,53].

Based on the filtered climatic variables, we identified three main types of potential suitable habitats for *Glipa*. First, the monsoon regions with high precipitation include large parts of east Asia, and certain areas of Mexico; and, second, the tropical rainforest regions comprise southeast Asia, the Amazon basin of South America, and the tropical rainforest region of central and western Africa. All of these regions are likely to be located in areas in which there is sufficient precipitation and heat for the optimal development of *Glipa* larvae and adults. Third, the Mediterranean region is typified by dry summers but cool and moist winters, with limited rainfall throughout the year. Because the winters are mild, we speculate that *Glipa* larvae in this region may complete their development during the relatively moist winters [54]. While phenological patterns have been documented in other insect groups—such as the scolytid beetle *Tomicus destruens* and the pine processionary moth *Thaumetopoea pityocampa* [55,56]—it remains unclear whether similar seasonal dynamics occur in *Glipa* spp. and how these patterns might affect their interactions with host plants.

### 4.2. Changes in the Potential Suitable Habitat of Glipa Under Future Climate Scenarios

Climate change has led to significant shifts in regional weather patterns and seasonal cycles.

The frequency and intensity of extreme weather events are also predicted to increase and significantly exacerbate the effects of habitat loss. This poses challenges to the current population sizes and distributions of many insects [57]. Some effects may even result in species extinction [58]. If the predicted temperatures are realized, the availability of floral resources for pollinators may decrease by 17–50% [59]. For example, the populations of insect pollinators in North America and Europe have already declined dramatically due to climate change [60,61,62]. However, some species may benefit if the suitable climate and habitat increase [63]. In response to such pressure, they may alter their geographical distribution through the expansion or contraction of suitable habitats and avert extinction [64]. There have been such changes in the distribution ranges of several insect species, such as Bornean moths and the mountain pine beetle (*Dendroctonus ponderosae* Hopkins) [65,66,67].

Our model concurs, at least in part, with some large-scale benefits of climate change for *Glipa.* The predicted distributions of suitable habitat are expected to expand towards higher latitudes, while retaining the original habitats. Such an expansion may result in *Glipa* maintaining or even increasing population sizes, thereby mitigating any negative effects of climate change. This prediction aligns with the projections from the CMIP5 and CMIP6 models, which forecast increased precipitation around the Mediterranean, eastern Europe, west-central Europe, Central America, and the northern part of northeastern North America in tandem with rising temperatures [68].

However, there may be negative effects associated with the predicted expansion of suitable habitats for *Glipa*. Host plants of *Glipa* include members of the Asteraceae and Apiaceae, such as fennel and wild radish [69]. It remains unclear how these plants and their suitable habitats will respond in the future to climate change. A spatial mismatch between host plants and suitable environmental conditions for *Glipa* may disrupt the plant–pollinator mutualistic relationships [70], the results of which are difficult to predict. However, two factors may be relevant to *Glipa*. First, after expanding to new habitats, *Glipa* could potentially disrupt, threaten, or even destroy the existing pollinator chain by expanding its niche to include other host plant species and competing with other species of insect herbivores and pollinators. This may result in changes in the community structure that may even result in the local extinction of host plants or other insect species [71]. Second, both the larvae and adults of *Glipa* are phytophagous. In the potential absence of natural enemies on the initial range expansion, *Glipa* populations may increase rapidly, thus posing a threat to host plants, including those of agricultural importance, in the new habitats. This study on the genus *Glipa*, which is known to contain 139 species, still has certain limitations. For instance, it relies on presence-only data. Moreover, given that the number of species with clear distribution point information is relatively limited in the actual research, the current distribution data are not comprehensive enough to accurately reflect the distribution ranges of each species. Therefore, in order to more comprehensively present the distribution of this genus, we chose to conduct a surrogate analysis at the genus level instead of listing the distribution points of each species individually [72]. Future research could incorporate additional potential influencing factors, such as the elevation, vegetation type, and human impact index, to improve the ecological realism and predictive accuracy of the model.

## 5. Conclusions

We predicted the centroid shift and the potential suitable habitat changes under various future climatic scenarios to provide a more comprehensive understanding of the impact of climate change on the distribution of *Glipa* spp. The maximum temperature of the warmest month, mean annual precipitation, and mean precipitation of the driest quarter were the three most important factors affecting the distribution of *Glipa*. We estimated the current total suitability area for *Glipa* as 2.33 × 10^7^ km^2^. Under future climate scenarios, this is expected to increase gradually as global temperatures continue to rise. In the context of global climate change, numerous insect pollinators face potential threats. Comprehensive studies should be conducted on more non-hymenopteran pollinating insects to understand more fully how they may respond to climate change, which would lead to a better understanding of how climate change affects pollinating insects in general.

## Data Availability

The original contributions presented in this study are included in the article/Supplementary Material. Further inquiries can be directed to the corresponding authors.

## Supplementary Materials

The following supporting information can be downloaded at: https://www.mdpi.com/article/10.3390/insects16060642/s1. Figure S1. The response curves of climatic factors. Table S1. Bioclimatic variables for Species Distribution Modeling. Table S2. Environmental variables and response outcomes used for modeling. Table S3. The suitable areas of *Glipa* under different climate scenarios (10^4^ Km^2^).
