# Supplementary material for: Predicting Suitable Habitat for Glipa (Coleoptera: Mordellidae: Mordellinae) Under Current and Future Climates Using MaxEnt Modeling
Source: Insects. 2025 Jun 18;16(6):642. doi: 10.3390/insects16060642 (PMC12194717; doi:10.3390/insects16060642)
Supplement: Supplementary file 1 [file insects-16-00642-s001.zip › insects-3619178-supplementary.pdf]

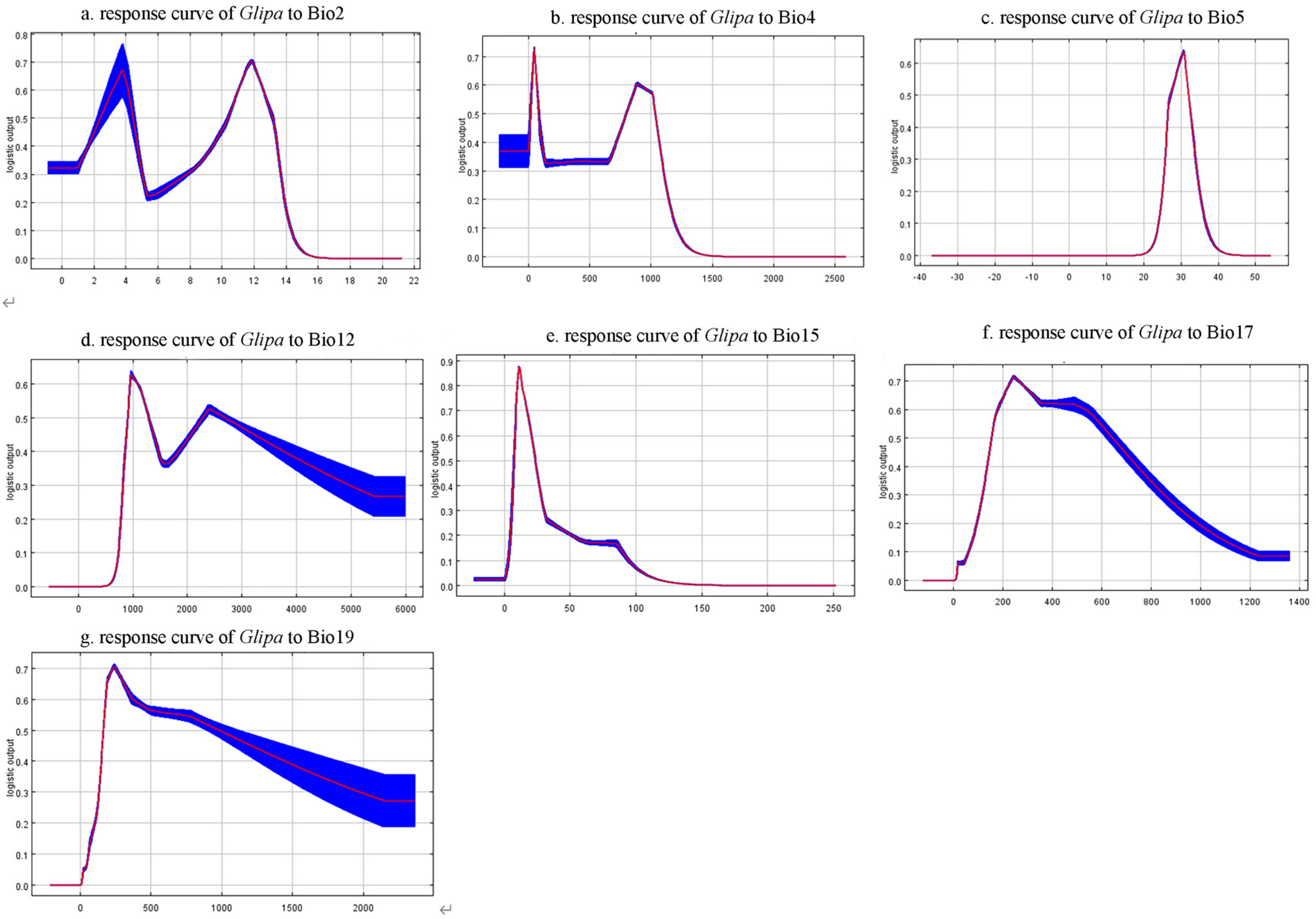

Figure S1. The response curves of Climatic factors

Table S1. Bioclimatic Variables for Species Distribution Modeling

Bioclimatic variables	Description
Bio1	Annual Mean Temperature
Bio2	Mean Diurnal Range (Mean of monthly (max temp - min temp))
Bio3	Isothermality (*100)
Bio4	Temperature Seasonality (standard deviation *100)
Bio5	Max Temperature of Warmest Month
Bio6	Min Temperature of Coldest Month
Bio7	Temperature Annual Range
Bio8	Mean Temperature of Wettest Quarter
Bio9	Mean Temperature of Driest Quarter
Bio10	Mean Temperature of Warmest Quarter
Bio11	Mean Temperature of Coldest Quarter
Bio12	Annual Precipitation
Bio13	Precipitation of Wettest Month
Bio14	Precipitation of Driest Month
Bio15	Precipitation Seasonality (Coefficient of Variation)
Bio16	Precipitation of Wettest Quarter
Bio17	Precipitation of Driest Quarter
Bio18	Precipitation of Warmest Quarter
Bio19	Precipitation of Coldest Quarter

Table S2. Environmental variables and response outcomes used for modeling.

Bioclimatic variables	Contribution (%)	Ranges of suitability	Most suitability bioclimatic values	Highest habitat suitability (logistic value)
Bio2	1.5	0-14.12℃	11.87℃	0.703
Bio4	8.7	-2.35-11.77	0.47/8.88	0.724
Bio5	14.9	24.52℃-35.84℃	30.64℃	0.634
Bio12	44.8	761.46-5982.9mm	970.32mm	0.628
Bio15	2.5	3.76-88.67	11.18	0.875
Bio17	27.1	78.12-1094.11mm	244.23mm	0.714
Bio19	0.6	73.89-2479.4mm	244.01mm	0.710

Table S3. The suitable areas of *Glipa* under different climate scenarios ($10^4 Km^2$)

Period	Climate scenario	The total Suitable habitat areas	Low Suitable habitat areas	Moderate suitable habitat areas	High suitable habitat areas
	Current	2333.38	1758.57	410.15	164.66
2030s	SSP126	2610.65	1997.90	435.21	177.54
	SSP245	2702.50	2039.34	434.52	228.64
	SSP370	2659.95	1985.63	409.97	264.35
	SSP585	2638.89	1980.68	387.86	270.35
2050s	SSP126	2754.19	2017.42	499.94	236.83
	SSP245	2918.42	2144.50	466.01	307.91
	SSP370	2868.36	2116.86	471.49	280.01
	SSP585	2970.69	2157.58	458.81	354.30
2070s	SSP126	2757.81	2021.77	451.34	284.70
	SSP245	2903.74	2144.50	466.01	293.23
	SSP370	3163.32	2286.61	508.21	368.50
	SSP585	3304.14	2363.26	480.25	460.63
2090s	SSP126	2716.83	1997.53	462.26	257.04
	SSP245	2957.12	2184.25	431.80	341.07
	SSP370	3365.61	2374.11	519.62	471.88
	SSP585	3590.85	2513.97	531.41	545.47
